# Exercise-Regulated Mitochondrial and Nuclear Signalling Networks in Skeletal Muscle

**DOI:** 10.1007/s40279-024-02007-2

**Published:** 2024-03-25

**Authors:** Elizabeth G. Reisman, John A. Hawley, Nolan J. Hoffman

**Affiliations:** Exercise and Nutrition Research Program, Mary MacKillop Institute for Health Research, Australian Catholic University, Level 5, 215 Spring Street, Melbourne, VIC 3000 Australia

## Abstract

Exercise perturbs energy homeostasis in skeletal muscle and engages integrated cellular signalling networks to help meet the contraction-induced increases in skeletal muscle energy and oxygen demand. Investigating exercise-associated perturbations in skeletal muscle signalling networks has uncovered novel mechanisms by which exercise stimulates skeletal muscle mitochondrial biogenesis and promotes whole-body health and fitness. While acute exercise regulates a complex network of protein post-translational modifications (e.g. phosphorylation) in skeletal muscle, previous investigations of exercise signalling in human and rodent skeletal muscle have primarily focused on a select group of exercise-regulated protein kinases [i.e. 5ʹ adenosine monophosphate-activated protein kinase (AMPK), protein kinase A (PKA), Ca2^+^/calmodulin-dependent protein kinase (CaMK) and mitogen-activated protein kinase (MAPK)] and only a small subset of their respective protein substrates. Recently, global mass spectrometry-based phosphoproteomic approaches have helped unravel the extensive complexity and interconnection of exercise signalling pathways and kinases beyond this select group and phosphorylation and/or translocation of exercise-regulated mitochondrial and nuclear protein substrates. This review provides an overview of recent advances in our understanding of the molecular events associated with acute endurance exercise-regulated signalling pathways and kinases in skeletal muscle with a focus on phosphorylation. We critically appraise recent evidence highlighting the involvement of mitochondrial and nuclear protein phosphorylation and/or translocation in skeletal muscle adaptive responses to an acute bout of endurance exercise that ultimately stimulate mitochondrial biogenesis and contribute to exercise’s wider health and fitness benefits.

**Supplementary Information:**

The online version contains supplementary material available at 10.1007/s40279-024-02007-2.

## Key Points

**Table:** 

Endurance exercise stimulates skeletal muscle signalling networks involved in the regulation of protein kinases and phosphorylation of protein substrates that underpin the benefits of exercise on health and fitness.
To date, skeletal muscle exercise signalling analyses have primarily focused on a select group of kinases and substrates. The recent applications of omics technologies have highlighted the complexity and interconnection of exercise signalling networks that involve mitochondrial and nuclear phosphoproteins.
By interrogating the existing literature and skeletal muscle exercise-regulated phosphoproteomic datasets, this review highlights signalling crosstalk and protein translocation between the mitochondrion and nucleus involved in stimulating mitochondrial biogenesis and eliciting exercise’s health benefits.

## Introduction

The health benefits of exercise training in delaying or preventing chronic diseases, such as obesity, insulin resistance, type 2 diabetes, cancer and heart disease, are widely accepted [1–5]. Exercise training remains one of the most accessible and effective interventions for the management and/or treatment of many lifestyle-associated chronic conditions. Understanding the complex biological networks and regulation underlying exercise-induced molecular adaptations can help frame exercise recommendations and identify future targeted therapies to promote the health benefits of exercise and enhance quality of life.

Exercise elicits whole-body physiological and molecular responses in numerous cells, tissues, and organs, such as skeletal muscle. To meet increased whole-body and tissue-specific energy demands during exercise, skeletal muscle contraction stimulates a network of cellular signalling pathways through the process of protein phosphorylation [6]. At the core of these exercise-regulated protein signalling responses are mitochondria, which play regulatory roles in maintaining cellular functionality as the main source of cellular energy conversion. Exercise-induced perturbations in mitochondrial homeostasis result in mitochondria sending numerous signals to reflect their metabolic status that can initiate a coordinated nuclear and cytosolic response (i.e. nuclear transcription and translocation) to promote cell-wide adaptations [7]. Maintenance of effective mitochondrial content and function to sustain adenosine triphosphate (ATP) synthesis has been previously linked to increased life expectancy. Various diseases are characterised by reduced mitochondrial content or disruption of multiple functions and behaviours of the mitochondria [8], such as cardiovascular diseases, cancer, obesity and type 2 diabetes [3, 9, 10]. To adapt to rapidly changing energy requirements in response to exercise, mitochondria are synthesised through the process of mitochondrial biogenesis via protein import machinery engaged through various interacting intracellular signalling networks.

Different modes of exercise stimulate divergent physiological and molecular responses within skeletal muscle. Typically, the term exercise broadly describes a vast range of physical activities that can be further categorised into subtypes, including endurance (EX) and resistance-based (REX) exercise. Each of these distinct modes of exercise are believed to engage unique networks of signalling pathways that ultimately elicit mode-specific physiological adaptations [11, 12]. For example, REX training induces improvements in strength and increases in muscle fibre cross-sectional area [13], whereas EX adaptations are characterised by fatigue resistance, enhanced oxidative capacity, increased mitochondrial density and mitochondrial protein content as a result of mitochondrial biogenesis [14–17]. Given the wide health-promoting adaptations that occur in response to EX and the critical roles of cellular signalling in mediating these biological processes, this review will primarily focus on human and rodent EX-induced mitochondrial and nuclear signalling networks, involving phosphorylation, that regulate skeletal muscle mitochondrial biogenesis.

### Exercise and Skeletal Muscle Energy Metabolism

Skeletal muscle tissue is composed of heterogeneous cell types and serves central roles in locomotion, thermoregulation, post-prandial glucose uptake and resting metabolic rate [18]. Exercise induces major homeostatic perturbations in numerous cell types within skeletal muscle. The adaptive responses of skeletal muscle to help meet these changing metabolic and contractile demands, such as during an acute bout of EX, are controlled by an intricate network of protein signalling pathways capable of stimulating a wide range of downstream biological responses. These coordinated responses stimulate a cascade of post-translational modifications (PTM), such as protein phosphorylation, that result in activation and/or deactivation of metabolic regulatory pathways that elicit a wide range of physiological, cellular and molecular adaptations to exercise [6, 19–22]. In addition to phosphorylation, other PTMs (e.g. acetylation, neddylation and ubiquitination) are also regulated by exercise via covalent bonds and other conformational changes at the substrate level.

The energy demands of exercise require a continual supply of ATP to maintain skeletal muscle contractile activity. EX results in a gradual increase in the adenosine monophosphate (AMP) to ATP ratio and the level of intracellular free calcium (Ca^2+^) which leads to activation of energy sensing and calcium responsive kinases and signalling pathways [23]. Muscle ATP demand increases with exercise intensity; ATP turnover can increase 100-fold above resting levels during maximal sprint exercise [6]. Utilising reducing equivalents that are generated from carbohydrate (CHO) and fat energy sources, these exercise-regulated signalling pathways facilitate substrate phosphorylation (anaerobic or oxygen-independent glycolysis), oxidative phosphorylation (OXPHOS) and the stimulation of a network of proteins that regulate gene transcription [24]. These transcripts are translated into precursor proteins for import into pre-existing mitochondria. Within the mitochondria, proteins either activate mitochondrial gene expression, act as specific subunit enzymes or are involved in the respiratory chain and substrate oxidation. This coordinated network of transcripts, proteins and signalling networks programs the expansion of the mitochondrial network in skeletal muscle and an increased capacity for the provision of aerobic ATP [25]. These signals must be transduced in reverse, starting from the nucleus to cytoplasm and then the mitochondria, to induce mitochondrial specific adaptive responses [7] that are central to enhancing skeletal muscle metabolic capacity and maintaining energy homeostasis in response to acute exercise and exercise training [26, 27].

### Exercise Mode, Intensity and Duration

‘Acute’ exercise describes a single session of exercise, whereas ‘chronic’ exercise training refers to repeated bouts of exercise that stimulate stable exercise-induced changes in skeletal muscle morphology and elicit physiological improvements in aerobic endurance capacity over time (i.e. weeks to months). Both the intensity and duration of an EX bout influence which signalling and downstream metabolic pathways become stimulated [28, 29]. Exercise intensity can be categorised into three broad types. High intensity interval exercise (HIIE) is defined as ‘near maximal’ efforts performed at an intensity between 85 and 95% of peak power output (*W*_max_), separated by intermittent rest or active recovery periods. Sprint interval exercise (SIE) is characterised by efforts performed at ‘supramaximal’ (all-out short burst) efforts separated by intervals of rest or active recovery. In contrast to HIIE and SIE, moderate intensity continuous exercise (MICE) describes prolonged, continuous exercise (> 30 min) performed at lower (60–85% of $\dot{V}$O_2_ max) intensities [29, 30]. The prevailing exercise intensity determines the relative contributions of fat and CHO fuel, leading to varying demands for ATP-generating capacity, thereby engaging different signalling networks to facilitate muscle cells’ intracellular responses to contractile stimuli and cellular energy stress [31, 32].

## Exercise-Regulated Cellular Signalling Networks

### Post-Translational Modifications-Phosphorylation

**Fig. 1:**
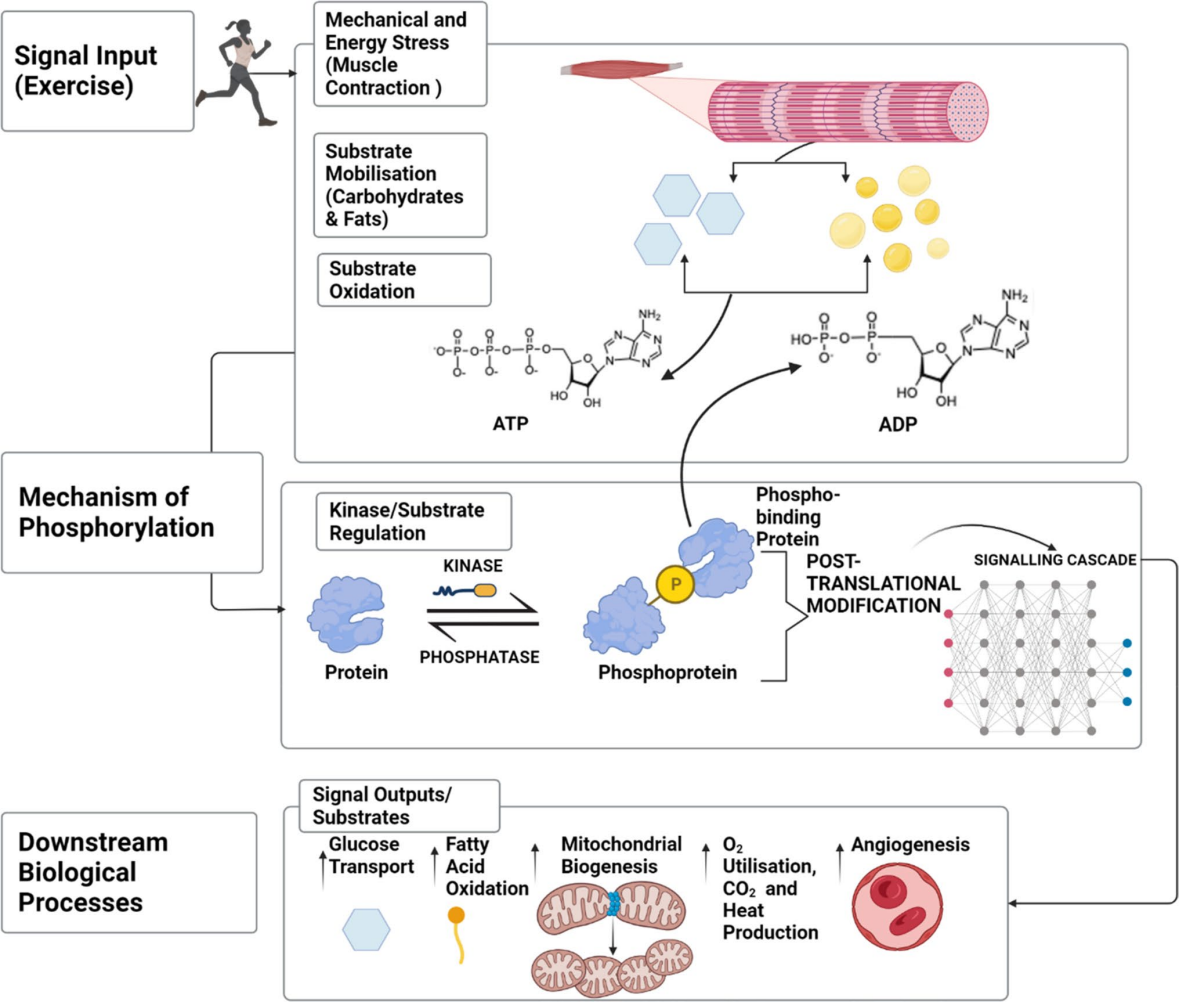
Exercise signal transduction networks and the mechanisms by which protein kinases initiate substrate phosphorylation and regulate downstream biological processes. The post-translational modification (PTM) of protein phosphorylation involves protein kinases, protein phosphatases and their respective substrate proteins. Phosphorylation is activated by cellular perturbations, such as skeletal muscle contraction and energy stress in response to exercise (i.e. signal input), which promotes energy substrate turnover and phosphorylation of target substrates capable of triggering a cascade of downstream signalling events. Consequently, substrate proteins receive a phosphate group via adenosine triphosphate (ATP) hydrolysis owing to enzymatic activity of the kinase. Phosphorylation is a reversible PTM owing to dual activity of kinases and phosphatases, which can allow them to serve as ‘molecular switches’ for their respective substrates’ phosphorylation and regulation of downstream biological processes. Created with BioRender.com

In response to metabolic and mechanical stress induced by contraction, coordinated cellular signalling networks become activated in skeletal muscle. Downstream physiological responses are influenced by the activation/deactivation and interactions occurring among proteins and genes within signalling networks that promote exercise-induced adaptations, such as mitochondrial biogenesis. In its simplistic form, signal transduction involves an enzyme (i.e. protein kinase) that phosphorylates its substrate protein(s) by transferring a phosphate group from ATP to the substrate protein via ATP hydrolysis (Fig. 1). The transfer of this large phosphate group influences the conformation and/or activity of the unique recipient substrate protein, for example through steric hinderance and electrostatic repulsion. This phosphorylation event can alter a protein’s function, molecular interactions and/or localisation to regulate downstream exercise-regulated biological processes.

There are many protein kinases regulated by EX in skeletal muscle, each capable of phosphorylating a specific subset of target substrate proteins and communicating with other kinases and interconnected signalling pathways [33, 34]. Reversible reactions involving protein phosphorylation by kinases and dephosphorylation by protein phosphatases within these signalling cascades can act as a ‘molecular switch’ to regulate protein kinase and downstream substrate activity to maintain homeostasis [34, 35], such as downstream regulation of transcriptional coactivators or transcription factors that influence gene expression networks involved in exercise-stimulated skeletal muscle mitochondrial biogenesis (Fig. 1). The extensive nature of phosphorylation events that can occur on metabolic enzymes illustrates the significance of phosphorylation as a key regulator of cellular metabolism [36] and most cellular biological processes [34], including the adaptation of skeletal muscle to acute EX and EX training.

### Key Exercise-Regulated Protein Kinases

Exercise elicits a wide range of protein phosphorylation events and other PTM. Previous investigations of these complex signal transduction networks primarily focused on a small and select group of exercise-regulated protein kinases. These widely studied kinases that become activated in response to exercise include the 5’ AMP-activated protein kinase (AMPK), protein kinase A (PKA), Ca2^+^/calmodulin-dependent protein kinase (CaMK), mitogen-activated protein kinase (MAPK) and mechanistic target of rapamycin (mTOR). AMPK, CaMK and MAPKs are recognised as major kinases activated by EX that help mobilise energy stores required to maintain muscle contraction and energy homeostasis during exercise, while mTOR is primarily activated by REX.

Historically, signalling pathways were mainly studied in a reductionist, hypothesis-driven manner by investigating individual or small subsets of kinases, substrates and pathways. However, an increasing number of exercise-regulated kinases and their respective pathways have been discovered; using recent technological advances, it has been possible to demonstrate that such pathways are not individual entities but parts of larger interconnected signalling networks [37]. This complexity and integrative nature of skeletal muscle exercise-regulated signalling networks provides compelling reasons to utilise more global, hypothesis-generating approaches to provide more in-depth investigations into how the wider breadth of signalling molecules can become activated or deactivated within their respective pathway(s), for example to stimulate mitochondrial biogenesis.

## Mitochondria—Core Hub of Skeletal Muscle Signalling and Energy Production

### Endurance Exercise as a Stimulus for Mitochondrial Biogenesis

Central to the EX signalling network in skeletal muscle are the mitochondria, which play pivotal roles in maintaining cellular functionality as the most rich and efficient cellular source of ATP utilised to meet increased energetic demands. Mitochondria are involved in the control of cellular energy substrate utilisation, calcium regulation, intracellular signalling coordination and programmed cell death [8, 38]. The production of new mitochondria in skeletal muscle in response to repeated EX training does not involve newly formed organelles within the cell, but instead is achieved via the assimilation of new protein components into the existing mitochondrial network [39]. Mitochondrial biogenesis is therefore typically recognised as the production of these new mitochondrial components [40, 41], the outcomes of which include alterations in mitochondrial quality (i.e. cristae density), respiratory function and/or content [42–44]. Mitochondria are comprised of both nuclear and mitochondrial genomes and as such, numerous signalling pathways converge on multifaceted regulatory processes to promote mitochondrial biogenesis. Apart from 13 proteins encoded by the mitochondrial genome that are inherited from the mother in most mammals [45], a substantial majority of mitochondrial proteins are encoded by nuclear DNA and therefore need to be dynamically imported into the mitochondria via the cytosol [46, 47].

**Fig. 2:**
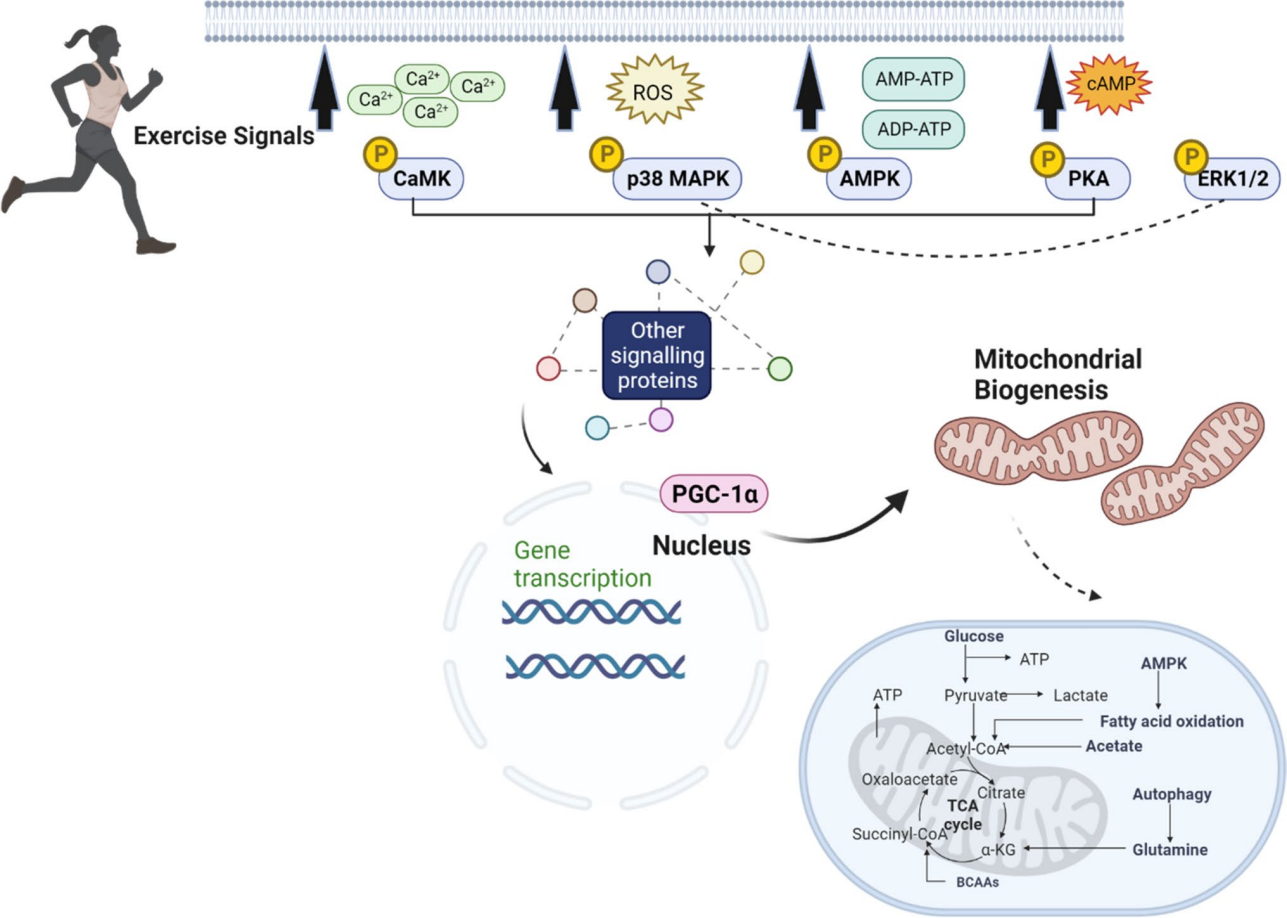
Schematic overview of upstream regulation of kinases and downstream signalling pathways in response to endurance exercise. Key upstream signals induced by a single bout of muscle contractile activity result in the activation of multiple kinases and downstream signalling pathways. A bout of endurance exercise generates intracellular upstream regulatory signals [e.g. calcium, reactive oxygen species (ROS), adenosine monophosphate/adenosine triphosphate (AMP/ATP) ratio, adenosine diphosphate/adenosine triphosphate (ADP/ATP) ratio and cyclic adenosine monophosphate (cAMP)] in skeletal muscle that activate kinases and promote downstream signalling and translocation of regulatory proteins, such as peroxisome proliferator-activated receptor-gamma coactivator 1-alpha (PGC-1α), to the nucleus. Increases in intracellular calcium are linked with the activation of calcium/calmodulin-dependent protein kinase (CaMK). The activation of AMP-activated protein kinase (AMPK), p38 mitogen-activated protein kinases (MAPK), extracellular signal-regulated kinases (ERK 1/2) and cAMP dependent protein kinase A (PKA) are also involved in the skeletal muscle signalling network activated in response to endurance exercise. This ultimately leads to increases in the transcription of genes in the nucleus involved in the regulation of mitochondrial biogenesis and adaptation of cellular metabolism in response to endurance exercise. Created with BioRender.com

EX training represents a potent stimulus for mitochondrial biogenesis by inducing homeostatic perturbations that affect nuclear and mitochondrial protein abundance and phosphorylation status. Mitochondrial function is tightly linked with the activation/deactivation of skeletal muscle signalling pathways through changes in cellular redox status and phosphate ratios, such as nicotinamide adenine dinucleotides (NAD/NADH), coenzyme Q (CoQH2/CoQ), ATP/AMP and adenosine diphosphate (ADP)/AMP levels in addition to metabolite (i.e. succinate, alpha-ketoglutarate and acetyl coenzyme A) and reactive oxygen species (ROS) levels (Fig. 2) [48]. Collectively, these responses are initiated by a unique sequence of intracellular signalling events, including the activation of exercise-responsive kinases and downstream signalling to their respective protein substrates, leading to subsequent increases in mitochondrial biogenesis [40, 49] (Fig. 2). Many kinases and phosphatases can localise to the mitochondria and play important cellular regulatory roles. However, the precise mechanisms by which these kinases and phosphatases translocate to mitochondria to regulate mitochondrial substrates, and the molecular and physiological consequences of their subcellular localisation, remain poorly understood [50]. Given that skeletal muscle mitochondria and nuclei represent key cellular hubs of signalling with central regulatory roles in health and athletic performance, the following section will summarise recent literature highlighting mitochondrial and nuclear-associated signalling pathways and PTMs involved in modulating gene expression and protein translation to stimulate skeletal muscle mitochondrial biogenesis.

### Mitochondrial Signalling Pathways and Protein Translocation

Mitochondrial quality control is governed by a range of biological processes, including mitochondrial biogenesis, fission, fusion, mitophagy and intracellular movement, to achieve mitochondrial homeostasis [48]. Mitochondrial biogenesis and fission processes provide cells with the ability to adjust the mitochondrial network to ultimately adapt to the prevailing cellular environment to combat mitochondrial damage and respiratory chain dysfunction [51]. Mitochondrial-to-nuclear communication to coordinate and regulate cellular processes is termed retrograde signalling; this transfer of information involves a global cellular response that ultimately modifies the expression level of various genes and proteins [48]. In the opposing direction, anterograde signalling coordinates the transfer of information from the nucleus and cytoplasm to the mitochondria [52–55]. Typically, retrograde responses are controlled by metabolic signals, such as mitochondria-related changes in intracellular Ca^2+^ dynamics, which result in changes in nuclear gene expression and adaptive alterations in metabolic and stress-related cellular pathways [56].

Signalling cascades are necessary for transducing information from the mitochondria to the nucleus to regulate the activation of transcription factors and subsequent transcriptional responses. While the exercise intensity-dependent regulation of substrate oxidation during EX is well established [57, 58], recent evidence implicates the regulation within mitochondria to be important to controlling substrate interactions [59] and PTMs involved in transport and oxidation of fats and CHOs. Specifically, exercise induced mitochondrial retrograde signalling may include changes in ROS, Ca^2+^, mitochondrial unfolded protein response, circulating metabolites and mitokines (i.e. molecules that signal local mitochondrial stress) levels. These may act directly to initiate a transcriptional response in the nucleus or via protein kinases, including AMPK, MAPK and CaMKs, and transcription factors, including mitochondrial transcription factor (TFAM), nuclear factor kappa-light-chain-enhancer of activated B cells (NF-κB,) and peroxisome proliferator-activated receptor gamma coactivator 1α (PGC1α) [7]. Evidence of mitochondrial signalling in response to acute EX is discussed subsequently.

### Nuclear Signalling Pathways and Protein Translocation

A major purpose of nuclear and mitochondrial signalling crosstalk is to achieve a well-developed and functional mitochondrial organelle system within the cell. Such signalling involving the nucleus is governed via protein translocation and involves the movement of numerous proteins to/from the nucleus to regulate a range of cellular events. Nuclear crosstalk and translocation of proteins between subcellular compartments helps to reduce cellular energy stress induced by exercise or regulate programmed cell death through the process of apoptosis. For example, proteins translocated from the nucleus redistribute to the cytosol and mitochondria at the onset of apoptosis [60]. The expression of mitochondrial nuclear-derived genes is therefore an important step in initiating the cellular program of mitochondrial biogenesis. As such, the bioenergetic capacity of skeletal muscle may ultimately be linked to the complex interplay of molecular processes activated by nuclear signalling proteins [61]. To date over 5000 proteins encoded by the human genome are annotated to reside in the nuclear compartment, and this likely represents just a small subset of the extensive array of possible signalling/translocation events and cellular adaptations that occur both at the mitochondrial interface and the nucleus.

Several PTMs control the nuclear versus cytoplasmic/mitochondrial localisation, including p53, a tumour-suppressor protein that functions as a nuclear transcription factor in response to cellular stressors to regulate gene transcription products. The accumulation of p53 in the nucleus is regulated by its phosphorylation, and the movement of p53 from the nucleus to the mitochondrial membrane is thought to be directed by phosphorylation of Bcl-2 homologous antagonist/killer (BAK) and Bcl-2-associated death (BAD) promoter [62]. Furthermore, forkhead transcription factor (FOXO3) contributes to the translocation of p53 to the mitochondria by increasing the association of p53 with the nuclear export machinery [62]. Such nuclear signalling regulation can also be observed via phosphorylation of proteins including sirtuin-1 (SIRT1) [63] and histone H3, which are involved in the regulation of gene expression and transcriptional activation, respectively. As detailed below, developing a more comprehensive blueprint of these interconnected proteins and PTMs, as well as roles of mitochondrial-nuclear protein translocation and signalling, may help uncover novel cellular signalling events underlying the central role of skeletal muscle mitochondrial biogenesis in health and performance [48].

### Current Understanding of Mitochondrial and Nuclear Signalling Networks Regulated by Acute Endurance Exercise

**Fig. 3:**
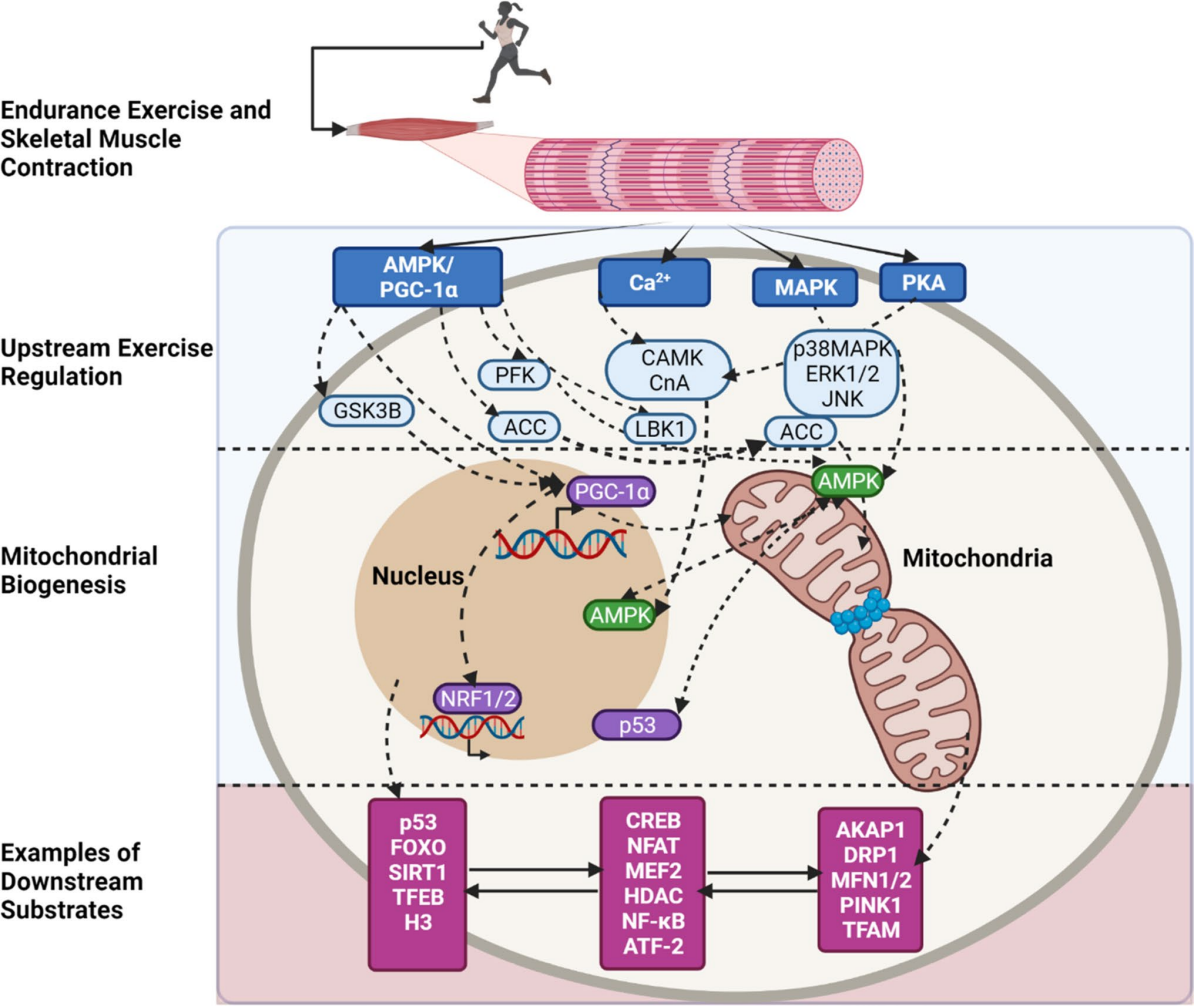
Schematic of the current endurance exercise-regulated molecular landscape of kinases and signalling at the mitochondrion and nucleus. Skeletal muscle contraction in response to a single bout of endurance exercise results in increased cellular energy demands. Exercise-stimulated activation of upstream and downstream regulated signalling events from the mitochondria to the nucleus are highlighted. AMPK and peroxisome proliferator-activated receptor-gamma coactivator 1-alpha (PGC-1α) are major regulators of mitochondrial biogenesis. Stress response regulators, such as adenosine monophosphate-activated protein kinase (AMPK), mitogen-activated protein kinases (MAPK), protein kinase A (PKA) and calcium-regulated signalling cascades, involve regulation of downstream substrates and biological processes including metabolism, apoptosis and mitochondrial dynamics related to mitochondrial biogenesis. Examples of common upstream exercise regulators and typical downstream substrates are highlighted. *AMPK* adenosine monophosphate-activated protein kinase, *PGC-1α* peroxisome proliferator-activated receptor-gamma coactivator, *MAPK* mitogen-activated protein kinases, *PKA *protein kinase A, *CnA* calcineurin, *CAMK* Ca^2+^/calmodulin-dependent protein kinase, *ERK* extracellular signal-regulated kinases, *JNK* c-jun N-terminal kinases, *PFK* phosphofructokinase, *GSK3B* glycogen synthase kinase 3 beta, *ACC* acetyl-CoA carboxylase, *LKB1* liver kinase B1, *AKAP1* A-kinase anchoring protein 1, *DRP1* dynamin-related protein 1, *MFN1/2* mitofusin-1/2, *PINK1* PTEN-induced kinase 1, *TFAM* mitochondrial transcription factor A, *CREB* cAMP responsive element binding protein 1, *MEF2* myocyte-specific enhancer factor 2, *NFAT* nuclear factor of activated T-cells, *HDAC* histone deacetylase, *NF-κB* nuclear factor kappa-light-chain-enhancer of activated B cells, *FOXO* forkhead transcription factor, *p53* tumour protein p53, *SIRT1* NAD +  − dependent deacetylase sirtuin-1. Created with BioRender.com

Evidence accumulated over the past decade has implicated mitochondrial- and nuclear-associated kinases and signalling processes in the adaptive muscle response to EX. Cellular stress responses induced by EX involve intracellular upstream regulatory signals (Fig. 2) and also sirtuin-mediated signalling, AMPK signalling or hypoxia-inducible factor 1-alpha (HIF-1α) signalling [64]. Kinases and signalling pathways commonly investigated in studies of EX include AMPK [65, 66], MAPK, CaMKII and PKA [25, 67], which converge to regulate biological processes, such as mitochondrial biogenesis. These commonly studied exercise-regulated kinases and downstream signalling proteins stimulated in response to acute EX are summarised in Fig. 3.

#### Regulation of Exercise-induced Mitochondrial Biogenesis by Peroxisome Proliferator-Activated Receptor-Gamma Coactivator (PGC-1α)

A single bout of EX generates intracellular signals that facilitate translocation of regulatory proteins, such as PGC-1α, to the nucleus, contributing to the coordinated regulation of nuclear and mitochondrial gene expression [51]. PGC-1α is a transcriptional coactivator that serves as a master regulator of mitochondrial content and function in skeletal muscle [68] and metabolic control in rat skeletal muscle [69]. Several studies in human skeletal muscle have shown that EX results in increased PGC-1α protein content within the nucleus [25, 67, 70, 71]. Cellular signalling cascades are the driving force underlying the activation of PGC-1α regulation by PTM and its direct phosphorylation by kinases [72]. While PGC-1α plays pivotal roles in the adaptive skeletal muscle response to exercise and modulation of mitochondrial biogenesis [73, 74], there exists a regulatory loop involving both PGC-1α and nuclear respiratory factor -2 (NRF2) [75, 76] driven by AMPK that regulates downstream PTM [77, 78]. AMPK can increase the level of NAD^+^ and stimulate a subsequent signalling cascade including SIRT1 phosphorylation [79]. When activated, SIRT1 deacetylates PGC-1α, and in turn promotes mitochondrial biogenesis, further phosphorylating p38 MAPK with increases up to two-fold following acute exercise [77, 80]. PGC-1α is also phosphorylated by glycogen synthase kinase 3β (GSK3β) (Fig. 3), inhibiting PGC-1α and thus influencing its intranuclear proteasomal degradation [81]. Additionally, there is an interactive layer of regulation between PGC-1α and transcription factor EB (TFEB) in which they translocate to the nucleus (mediated by the phosphatase calcineurin) and induce the expression of genes involved in mitochondrial biogenesis to augment mitochondrial substrate utilisation in response to exercise [82, 83]. Furthermore, the exercise-induced increase in mitochondria is a consequence of PGC-1α activation with a subsequent increase in its protein content that mitigates the increase in mitochondrial biogenesis. At rest, skeletal muscle PGC-1α is found predominantly in the cytosol, with its movement into the nucleus activated by exercise-induced activation of the p38 MAPK signalling pathway [84].

PGC-1α’s abundance in skeletal muscle is regulated by the intensity and duration of acute EX. The intensity-dependent regulation is mediated by differential activation of multiple signalling pathways. The phosphorylation of activating transcription factor 2 (ATF-2) and histone deacetylase (HDAC) act as pivotal mediators of EX intensity-dependent regulation of PGC-1α [85]. EX duration can increase PGC-1α protein content up to three-fold above rest following prolonged exercise [86], with increases of a similar magnitude after a repeated EX bout that ultimately stimulate mitochondrial biogenesis [87].

#### AMPK and Calcium Signalling Pathways Regulating Mitochondrial Function and Biogenesis

AMPK is one of the most widely studied exercise-regulated kinases, functioning as a sensor of cellular energy status to help maintain cellular homeostasis [88]. When cellular energy levels decrease in response to exercise (i.e. increased AMP/ATP and ADP/ATP ratios), AMPK becomes activated and phosphorylates its downstream substrates, thereby activating ATP-generating pathways (e.g. glucose transport and fat oxidation) while inhibiting pathways that consume ATP (e.g. fat and protein synthesis). AMPK’s influential role in mitochondrial function and homeostasis is well established [89]. Additionally, CaMK is controlled by multi-site phosphorylation and becomes activated in response to increases in intracellular calcium concentration upon muscle contraction owing to release of sarcoplasmic reticulum (SR) Ca^2+^ stores [90, 91]. As such the role of calcium signalling with exercise is linked to the modulation of ion homeostasis, CHO metabolism and gene transcription [92, 93].

AMPK targets an array of cellular substrates that regulate energy utilisation and storage pathways. AMPK mobilises from the cytoplasm to the nucleus and mitochondria, sensing energy demands to help maintain energy homeostasis by phosphorylating its substrates residing at these subcellular compartments [94, 95]. In human skeletal muscle, AMPK phosphorylation increases during both moderate and high intensity EX in humans [96, 97]. Along with its counterpart, PGC-1α, evidence suggests that AMPK is a major regulator of mitochondrial biogenesis in skeletal muscle [75, 98, 99]. When the mitochondria become the main cellular source for energy during exercise, energy-sensing proteins, such as AMPK, in the cytosol signal this cellular information to its substrates to modulate mitochondrial function and dynamics [100–102].

Other classical targets of AMPK that contribute to its regulation of mitochondrial metabolism include acetyl-CoA carboxylase (ACC) and 6-phosphofructo-2-kinase (PFK-2) [98, 103, 104] (Fig. 3). Two isoforms of ACC have been identified, ACC1 and ACC2, with distinct organelle distribution and regulation. ACC1 is cytosolic whereas ACC2 is associated with the mitochondria. ACC plays pivotal roles in the regulation of mitochondrial fatty acid oxidation and synthesis [105] when phosphorylated by AMPK in the cytoplasm [106]. ACC phosphorylation increases with increasing exercise intensity [85, 97, 107] and is dependent on endogenous substrate availability during exercise. ACC phosphorylation is two-fold higher when exercise is commenced with low (163 mmol/kg) compared with high (909 mmol/kg) muscle glycogen, provoking an enhanced uptake of fuels in glycogen-depleted conditions [108–110]. Accumulating evidence suggests that AMPK acts as a sensor of glycogen stores via key sites that mediate glycogen binding located within AMPK’s β subunit CHO-binding module, which can affect exercise capacity and substrate utilisation when these sites are disrupted in mice [111–113].

Recently, isolated mitochondrial fractions from exercised and/or contraction-stimulated mouse skeletal muscle have detected specific AMPK isoforms localised to the outer mitochondrial membrane and reported subcellular specific responses of AMPK. This pool of mitochondrial targeted AMPK serves as a bioenergetic sensor to attenuate exercise-induced mitophagy in skeletal muscle [114]. Recent evidence has identified a novel mechanism of mitochondrial biogenesis following mitochondrial disruption by which folliculin-interacting protein 1 (FNIP) phosphorylation is required for AMPK to activate TFEB and increase PGC-1α abundance [115]. The breadth of molecular mechanisms underlying AMPK-dependent regulation of subcellular metabolic processes involved in energy consumption and storage in response to exercise is an exciting area for future research.

Calcium-regulated signalling pathways and kinases are important for maintaining intracellular calcium homeostasis, including the regulation of Ca^2+^ uptake into the mitochondria. Conversely, Ca^2+^ has also been reported to diffuse from the cytosol to the nucleus. Two well characterised pathways of transcriptional activation include signalling through calcineurin A (CnA) and calmodulin-dependent protein kinase IV (CaMKIV), which interacts with PGC-1α by activating myocyte enhancer factors [116]. CaMKIV further activates PGC-1α by phosphorylating the transcription factor cyclic adenosine monophosphate (cAMP) response element (CRE)-binding protein (CREB) (Fig. 3) [117]. Meanwhile, CaMKII signalling increases two-fold with increasing exercise intensity [85]. Additionally, liver kinase B1 (LKB1) and CaMKII [118] serve as upstream kinases for AMPK [119, 120]. The predominantly nuclear-localised kinase LKB1 regulates contraction-stimulated glucose transport in skeletal muscle with AMPK [118]. Increases in AMP binding to AMPK associated with the induction of cellular energy stress during EX leads to conformational changes in AMPK, making it a better substrate for LKB1 [13, 103]. While based in the nucleus, the translocation of LKB1 has been suggested to play a critical role in maintaining basal levels of mitochondrial enzymes [121]. These examples of AMPK and calcium signalling pathways underpinning mitochondrial biogenesis further highlight the interrelation and complexity of signalling networks and clearly warrant more global, unbiased approaches to uncover signalling pathways that become activated or deactivated in skeletal muscle response to acute EX.

#### PKA Signalling and Regulation of Mitochondrial Function and Dynamics

PKA is also a major regulator of skeletal muscle mitochondrial dynamics and exercise training-induced adaptations through its phosphorylation of a number of cellular targets [122]. EX stimulates glycogen breakdown via β-adrenergic receptors, which subsequently leads to accumulation of intracellular cAMP and activates PKA and its downstream signalling pathway [123]. Several observations link this pathway with several components of metabolism following exercise. cAMP has been shown to increase up to two-fold following the onset of EX, a response dependent on muscle fibre type, duration and intensity [124]. There is evidence that PKA localises to the mitochondrial matrix and the inner mitochondrial membrane and influences signalling to the mitochondria [125, 126]. The subcellular localisation of PKA is thus important for PKA-dependent downstream signalling. Recent studies have shown that A-kinase anchor protein 1 (AKAP1) recruits PKA and other signalling proteins to localise to the outer mitochondrial membrane, thereby integrating several second messenger cascades by inhibitory phosphorylation of mitochondrial fission regulator dynamin-related protein 1 (DRP1; Fig. 3) [127]. Furthermore, following EX the inhibition of PKA leads to significant elevation in MAPK exercise-responsive kinase group extracellular signal-regulated kinases 1 and 2 (ERK1/2) signalling [128, 129]. These stress kinase responses to exercise are discussed subsequently.

#### MAPK Signalling and Regulation of Exercise Induced Mitochondrial Biogenesis

MAPKs are stress kinases that are activated in response to mechanical stress (i.e. muscle contraction during EX) and control numerous biological processes including regulation of metabolism and downstream modulation of gene expression [130]. To date MAPKs have three main exercise responsive kinase groups, including ERK1/2, c-Jun amino-terminal kinases (JNKs) and p38 isoforms [131, 132]. MAPK signalling through the ERK1/2 and p38 MAPK pathways increases up to seven-fold immediately post EX [130, 133, 134], with evidence linking MAPK signalling cascades to downstream regulation of skeletal muscle gene expression in response to EX [128].

MAPK signalling pathways serve key functions in exercise-induced skeletal muscle adaptations, maintenance of whole-body glucose homeostasis and ROS production [130, 135]. Key ROS-sensitive protein kinase signalling responses induced by EX include JNK, p38 MAPK, and nuclear factor kappa-light-chain-enhancer of activated B cells (NF-κB). The p38 MAPK and JNK signalling pathways play important roles in exercise-stimulated mitochondrial biogenesis (Fig. 3) [80]. Exercise also results in increased, but transient, ROS production, contributing to activation of MAP kinases in skeletal muscle [136]. The effects of acute HIIE and SIE on the activation of these signalling pathways remain unclear. However, SIE elicits greater skeletal muscle NF-κB phosphorylation immediately after exercise compared with HIIE, with JNK and p38 MAPK phosphorylation [130, 137] increasing to a similar magnitude independent of exercise intensity [137]. Studies have further linked the activation of AMPK to the downstream phosphorylation of p53, which can be a target of p38 MAPK under conditions of cellular stress [138]. Bartlett et al. [139] demonstrated exercise-induced p53 phosphorylation in humans, noting that both acute HIIE and MICE stimulate increases in AMPK and p38 MAPK phosphorylation in skeletal muscle.

While whole skeletal muscle analyses have demonstrated increases in p53 phosphorylation, detection of specific muscle subcellular fractions will broaden our understanding regarding the nuclear protein accumulation of p53 and its translocation to other cellular compartments. This pathway is known to be predominantly facilitated by PTM controlling nuclear/cytosolic p53 shuttling and stabilisation within the nucleus [140]. p53 protein stabilisation and accumulation within the nucleus was demonstrated following EX in humans, while its mitochondrial content remained unchanged [141]. Subcellular analyses have investigated signalling in nuclear and cytosolic fractions in response to different types of exercise, revealing that p38 MAPK increased four-fold in the nuclear fraction immediately following SIE but not following MICE [142]. These findings from human and rodent muscle have primarily focused on a specific subset of kinases and/or substrates and highlight the advantages of using subcellular fractionation to increase protein and phosphorylation site identification within specific subcellular compartments. While the results of these studies provide evidence of intracellular protein localisation and translocation in response to EX, the application of targeted approaches focussing on only a panel of phosphorylated and/or total proteins of interest in isolation highlights an existing gap in our understanding of how the nuclear and mitochondrial proteome responds to EX to promote mitochondrial biogenesis. As a result, global omics-based approaches represent a promising avenue to improve the accuracy and depth of identification and quantification of these complex skeletal muscle exercise-regulated subcellular signalling networks.

## Omics-Based Approaches to Map Exercise-Regulated Signalling Networks

### Phosphoproteomics

**Fig. 4:**
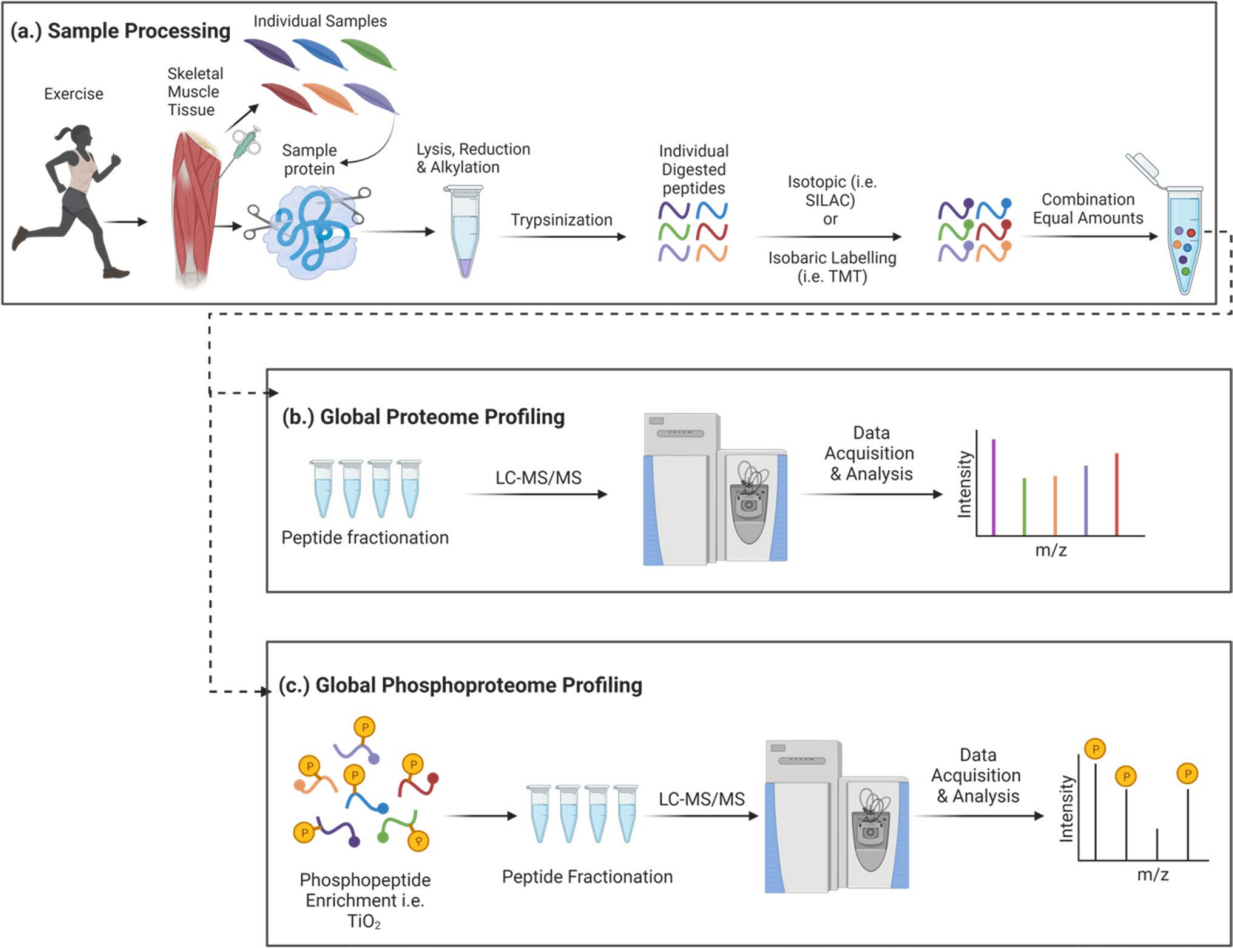
Proteomic and phosphoproteomic sample preparation and analysis workflow overview. **a** In MS-based phosphoproteomic analysis workflows, proteins are first homogenised and extracted from skeletal muscle samples. Following protein extraction, the sample preparation workflow then involves protein extraction, denaturing, reduction and alkylation followed by trypsin digestion into peptides. Isotopic [i.e. stable isotope labelling by amino acids in cell culture (SILAC)] or isobaric labelling [i.e. tandem mass tag (TMT)] of peptides is commonly performed to improve the sensitivity and throughput of sample analyses by combining tagged samples, whereby labelled peptides from each sample are combined in equal amounts and can be distinguished from each other by MS; **b** this combined labelled sample can then be further fractionated to reduce the overall sample complexity; and **c** in global phosphoproteome profiling, phosphopeptides are enriched [e.g. using titanium dioxide (TiO_2_)] and fractionated with liquid chromatography (LC) before being identified and quantified by LC–MS/MS analysis. Finally, total peptides and/or phosphopeptides are identified by their mass to charge ratios (*m*/*z*) and phosphorylation site positions are assigned [178]. Adapted from ‘global protein and phospho profiling’, by BioRender.com (2023). Retrieved from https://app.biorender.com/biorender-templates

Omics-based approaches involve the large-scale study of genes, peptides, proteins, PTMs (such as phosphorylation), metabolites, and lipids in each biological system or tissue, such as skeletal muscle. Highly sensitive mass spectrometry (MS)-based technologies permit a global, unbiased approach to identify/quantify protein composition and phosphorylation status using proteomics and phosphoproteomics, respectively, to map skeletal muscle protein composition and signalling machinery in a manner unparalleled by most other technologies. MS-based proteomics and phosphoproteomics are therefore becoming indispensable tools to map the global skeletal muscle signalling responses to exercise and training adaptations. Recent technological advances in MS-based phosphoproteomic profiling permit unprecedented global investigations of the breadth of protein phosphorylation events and signalling network complexity. It is estimated that up to 50% of all proteins become phosphorylated during their lifetime [143]. Measuring protein phosphorylation requires the enrichment of phosphopeptides. This enrichment is typically achieved via the negatively charged phosphate group on a phosphopeptide preferentially binding to positively charged metal ions prior to MS analysis [144], enabling phosphopeptide identification/quantification and phosphosite localisation in an unbiased manner to map how proteins are transiently regulated, for example in response to acute exercise [145] (Fig. 4).

Semi-quantitative immunoblot analysis is commonly employed to measure the phosphorylation status of target protein(s) in a homogenised sample (e.g. skeletal muscle protein lysate) using antibodies that recognise specific amino acid residue(s) modified with a phosphate group. However, these types of a priori target analyses fail to provide detailed information and quantification of the wider breadth of known and novel phosphorylation sites potentially regulated within a protein of interest [146]. Following decades of technological advancements to develop more global, unbiased strategies to map complex signalling networks, phosphopeptide enrichment and MS-based phosphoproteomics enable simultaneous site-specific identification and quantification (i.e. fold-change in the intensity of a wider range of identified phosphosites between samples/conditions) of thousands of distinct phosphorylation events that can potentially regulate protein functionality and ultimately stimulate skeletal muscle adaptations, such as mitochondrial biogenesis in response to EX. The most commonly used phosphopeptide enrichment strategies include immobilised metal affinity chromatography and metal oxide affinity chromatography owing to their high selectivity and sensitivity (Fig. 4) [147]. Exercise signalling networks comprise coordinated and interconnected signalling events involving many kinases and substrates, with many features of these complex and dynamic exercise signalling networks typically overlooked by targeted approaches that repeatedly analyse the same subsets of kinases and substrates. However, the high associated costs and limited access to MS technologies present challenges to widely employing these global approaches. There is value in using both targeted and global approaches when mapping and interrogating these signalling networks, as determining the functional significance of potential novel protein phosphosites within the wealth of data acquired by MS will still most likely require further targeted validation approaches, such as site-directed mutagenesis, immunoblotting and experiments specifically testing functional biological readouts of these mutations.

The large dynamic range of protein abundance (i.e. low abundance proteins to highly abundant contractile proteins) in skeletal muscle is particularly challenging when characterising the proteome and its PTMs in response to exercise. These highly-abundant contractile proteins represent approximately 60% of total protein within human skeletal muscle [e.g. the largest protein being titin [molecular weight: three million Dalton)], in contrast to mitochondrial proteins that comprise approximately 28% of total human skeletal muscle protein content [148]. Given the central role of mitochondrial signalling and nuclear–mitochondrial crosstalk in maintaining energy homeostasis within skeletal muscle, there has been a recent growth in the application of technological approaches to study protein translocation and PTM regulation between different subcellular compartments. The development of techniques for selective enrichment of phosphopeptides from complex samples, as well as optimised subcellular fractionation, MS sample preparation and bioinformatic workflows, have collectively expanded coverage of the mitochondrial proteome/phosphoproteome with the number of identified mitochondrial protein phosphorylation sites increasing by orders of magnitude using these approaches [149, 150]. For example, the mitochondrial proteome is now more fully characterised, with 1136 recognised proteins [151] and 1038 of these with at least one experimentally determined phosphosite [150]. Furthermore, the use of subcellular fractionation to isolate specific cellular organelles of interest, such as mitochondria and nuclei, is a potential method to help reduce the overall skeletal muscle sample complexity and increase phosphorylation site identification within a specific subcellular compartment of interest. The recent developments and applications of phosphoproteomics utilising high-sensitivity MS has contributed to a rapid rise in the number of identified cellular kinases, substrates and regulatory phosphorylation sites associated with the regulation of mitochondrial biogenesis. While there remains uncertainty around the specific kinases and phosphatases involved in mitochondrial biogenesis and their functional importance in mitochondrial signalling pathways [149], an extensive study of tissue specific phosphorylation across rat tissues detected 336 phosphorylation sites in skeletal muscle mitochondria [152] with important links to pathways involved in ATP synthesis and the mitochondrial respiratory chain. Limitations still exist in identifying the numerous forms of proteins that may exist and be post-translationally modified (i.e. proteoforms which have specific amino acid sequences and PTMs that require different workflows to detect, such as top-down proteomics).

### Phosphoproteomic Analyses of Endurance Exercise-Regulated Signalling Networks

To date the limited phosphoproteomic-based studies of acute EX signalling networks have been restricted to mapping responses in whole skeletal muscle samples collected before and after a single bout of cycling in humans, treadmill running in mice/rats and in situ muscle contraction in rats [153–155]. The first study of the human skeletal muscle exercise-regulated phosphoproteome [154] identified > 8500 phosphorylation sites on 4317 proteins in four healthy male participants pre- and immediately post-exercise (i.e. moderate to high intensity cycling until volitional fatigue). Novel phosphosites (> 900) and 45 protein kinases were annotated to regulate at least one exercise-responsive substrate phosphorylation site. Additionally, this study functionally validated A-kinase anchoring protein 1 (AKAP1) as a mitochondrial AMPK substrate while demonstrating novel roles for AMPK-mediated phosphorylation of AKAP1 in regulating mitochondrial respiration. A subsequent study [153] in skeletal muscle following a single bout of treadmill running in mice and in situ rat muscle contraction quantified 1752 significantly regulated phosphosites by exercise in vivo and 1887 by in situ contraction, of which only 7 were annotated as mitochondrial proteins. When these datasets were compared with the previous analysis of human skeletal muscle exercise signalling [154], there was a high degree of convergence for key protein kinases regulated between these cross-species exercise and muscle contraction models (i.e. AMPK, S6K, PHKA1, CAMK and mTOR) [153]. However, these cross-species models showed a low degree of overall overlap for exercise-regulated phosphosites (67 phosphosites), with only AMPK and PKA observed as the two kinases significantly enriched in pathway analyses across all three exercise models.

The latest study in human skeletal muscle [156] highlights the potential of phosphoproteomic analysis to uncover both common and unique signalling pathways in response to different exercise modalities (i.e. EX, SIE and REX). Using a crossover study design 5486 phosphosites regulated during or after at least one exercise modality were identified, with 420 core phosphosites common to all three exercise modalities. The uncharacterised protein C18ORF25 was also discovered to be a novel canonical substrate of AMPK [156]. The three exercise modalities induced similar levels of phosphorylation of AMPK and its downstream substrate ACC1 immediately following exercise. While MAPK activated protein kinase 2 (MAPKAPK2) activation was increased across all modalities immediately post exercise, it was suppressed 3 h into recovery. Similarly, PKA’s level of activation increased, although its response was higher in SIE compared with EX.

A recent study in mice [157] employed phosphoproteomics to characterise signalling pathways modulated by high-intensity EX undertaken at different times of day, identifying approximately 1600 unique phosphoproteins. Catabolic and stress-related pathways had greater enrichment of protein phosphorylation events following early night-time [zeitgeber time (ZT) 12] versus early daytime exercise (ZT0) immediately after and 3 h post-exercise. Links to mitochondrial and nuclear signalling were observed with the identification of exercise regulated kinases, such as AMPK and unc-51-like autophagy activating kinase 1 (ULK1), which are known to translocate to the mitochondria [158]. This dataset provides the first global map of the distinct diurnal responses to HIIE in mice and an indication of possible mitochondrial and nuclear signalling in response to mouse treadmill exercise [157]. Amongst the global phosphoproteomics-based approaches to date in human and rodent skeletal muscle, two of the most enriched signalling pathways in response to EX are the AMPK and Ca^2+^-regulated signalling pathways highlighted in Sect. 3.4.2 [153]. Global phosphoproteomic analyses of skeletal muscle exercise signalling networks will continue to expand understanding of the complexity of AMPK-dependent phosphorylation events and the regulation of diverse cellular metabolic pathways by AMPK and other kinases in response to exercise [159].

### Skeletal Muscle Exercise-Regulated Mitochondrial and Nuclear Annotated Phosphoproteins from Existing Phosphoproteomic Datasets

**Fig. 5:**
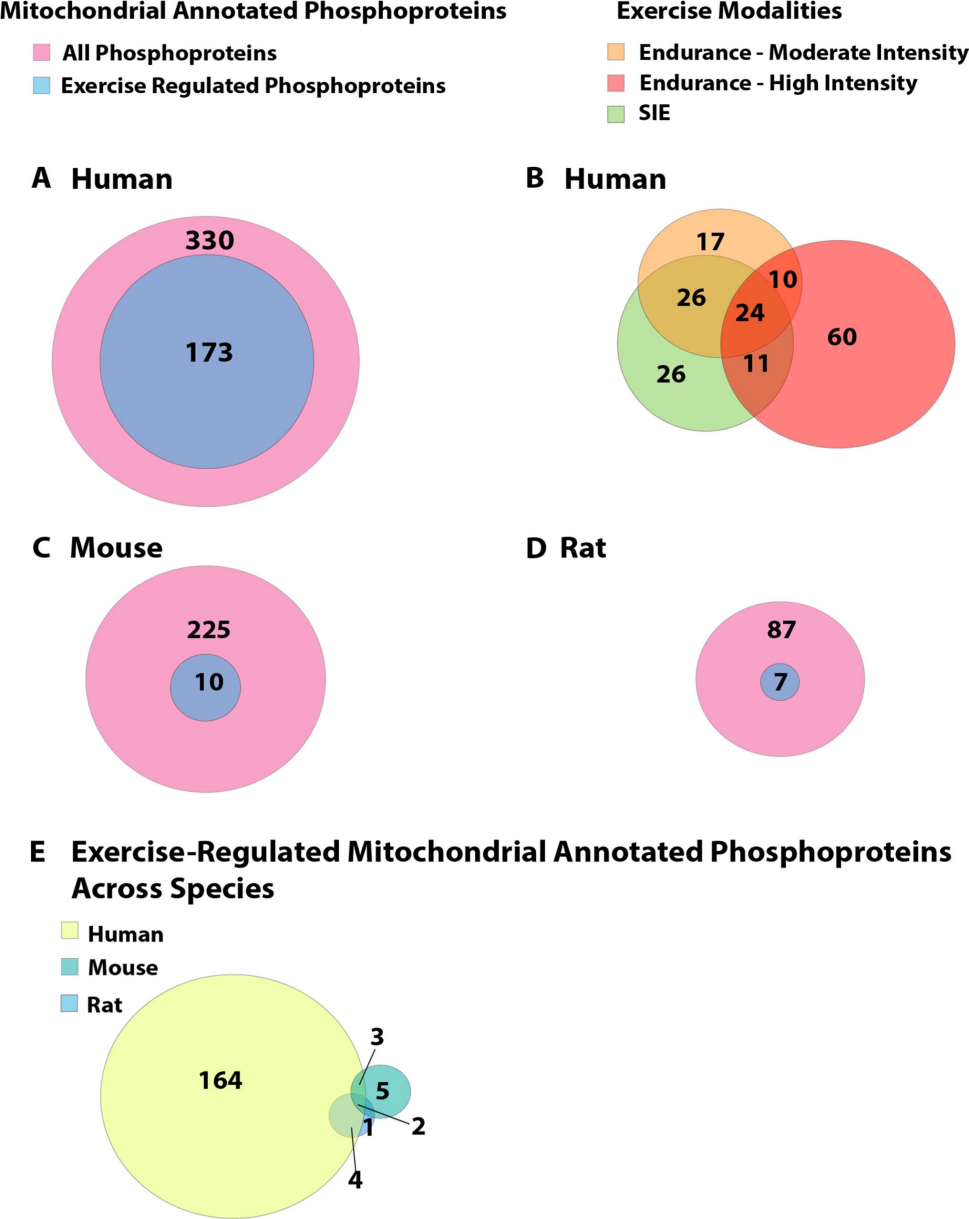
Venn diagram displaying the subcellular analyses of mitochondrial-annotated and exercise-regulated proteins identified in existing exercise and skeletal muscle phosphoproteomic databases [153, 154, 156, 157]. Data from each study were first filtered for only significantly regulated phosphoproteins (adjusted *P* or* Q* value < 0.05) and then assigned to their respective gene names within each dataset. These gene names were then imputed into database for annotation, visualization and integrated discovery (DAVID) software (51) for gene ontology cellular component (GOCC) analysis (with the analysis performed for each individual species) to identify common and/or unique exercise-regulated genes corresponding to their respective phosphoproteins in human skeletal muscle [154, 156] (**A**); human skeletal muscle across three different exercise modalities [moderate intensity EX, 60% peak oxygen uptake ($\dot{V}$O_2_ peak)] and sprint interval exercise (SIE) [156] and high intensity EX [85–92% peak power output (*W*_max_)] [154] (**B**); treadmill or wheel running in mice [153, 157] (**C**); in situ muscle contraction in rats [153] (**D**); and across all three species (**E**) that are annotated to reside at the mitochondrion. Phosphoproteins from all comparisons shown in Fig. 5 are listed in Supplementary Table S1

Signalling and crosstalk between the mitochondria and nucleus are important processes of skeletal muscle responses to acute exercise. To determine the breadth of exercise-regulated subcellular signalling events identified to date, we performed compartmental analyses of mitochondrial and nuclear phosphoproteins annotated to reside in these subcellular compartments using the existing exercise phosphoproteomic datasets described previously [153, 154, 156, 157]. The aim of these subcellular analyses was to identify the overall coverage, as well as potential unique and conserved exercise-regulated mitochondrial and/or nuclear phosphoproteins, within these phosphoproteomic datasets and distinguish potential differences between exercise modalities and across species. Post-hoc analysis revealed substantial differences in the total number and composition of mitochondrial phosphoproteins (corresponding to their respective gene names) regulated by exercise between human and rodent skeletal muscle (Fig. 5, Supplementary Table S1). In human skeletal muscle, exercise differentially regulated the phosphorylation of approximately half of the proteins annotated to the mitochondria (Fig. 5A; i.e. 173 of 330 total) compared with only ~ 4–8% of phosphosites regulated by exercise or muscle contraction in mice and rats, respectively (Fig. 5C, D; i.e. 10 of 225 total and 7 of 87 total, respectively). Skeletal muscle mitochondrial content, as well as muscle fibre type composition [160], differ between humans and rodents [161, 162]. As such, the observed variability in the proportion of exercise-regulated phosphoproteins amongst the total proteins annotated to reside at the mitochondria may be a consequence of species differences, sex differences [153] and/or variables associated with the specific exercise intervention in each species [153, 154, 156].

When further examining potential differences in skeletal muscle samples across the three exercise modalities in humans that have utilised a similar MS sample preparation workflow [154, 156], more mitochondrial annotated phosphoproteins were observed to be exercise-regulated by moderate and high-intensity EX compared with SIE (Fig. 5B). These data suggest that exercise volume rather than intensity may be a more potent stimulus for subcellular mitochondrial signalling, as well as potentially mitochondrial biogenesis, in response to exercise. Further interrogation of mitochondrial protein overlaps between species found only two common mitochondrial-annotated phosphoproteins (mTOR and plectin; PLEC) regulated by exercise across all three species using GOCC annotation (Fig. 5E). Only three phosphoproteins overlapped between humans and mice [prohibitin-2; PHB2 and ADP/ATP translocase; solute carrier family 25 member 4 (SLC25A4), damage specific DNA damage-binding protein 1 (DDB1) and cullin-4A (CUL4) associated factor 8; (DCAF8)] and four mitochondrial phosphoproteins overlapped between humans and rats (nitric oxide synthase; NOS1, mitochondrial fission factor; MFF, A-kinase anchoring protein 1; AKAP1, voltage-dependent anion-selective channel protein 1; VDAC1), while only two proteins were shared between rodent species (mTOR and PLEC; Fig. 5E). As demonstrated by these post-hoc subcellular analyses of publicly available phosphoproteomic datasets, there are many more mitochondrial-annotated proteins likely regulated by exercise yet to be discovered.

**Fig. 6:**
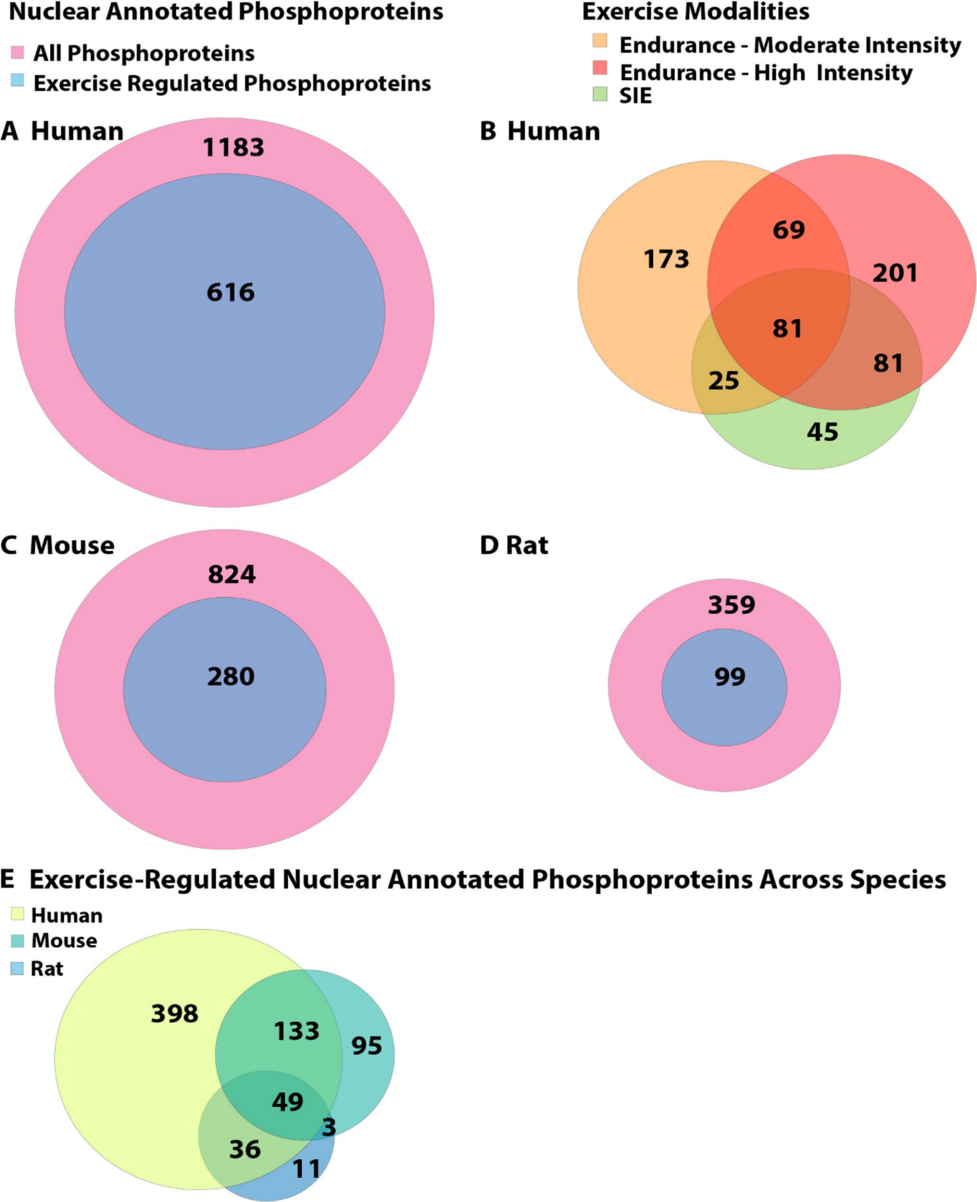
Venn diagram displaying subcellular analyses of nuclear-annotated and exercise-regulated proteins identified in existing exercise and skeletal muscle phosphoproteomic databases [153, 154, 156, 157]. Data from each study were first filtered for only significantly regulated phosphoproteins (adjusted *P* or* Q* value < 0.05) and then assigned to their respective gene names within each dataset. These gene names were then imputed into database for annotation, visualization and integrated discovery (DAVID) software (51) for gene ontology cellular component (GOCC) analysis (with the analysis performed for each individual species) to identify common and/or unique exercise-regulated genes corresponding to their respective phosphoproteins in human skeletal muscle [154, 156] (**A**); human skeletal muscle across three different exercise modalities [moderate intensity EX, 60% peak oxygen uptake ($\dot{V}$O_2_ peak) and sprint interval exercise (SIE) [156] and high intensity EX (85–92% peak power output (*W*_max_)] [154] (**B**); treadmill or wheel running in mice [153, 157] (**C**); in situ muscle contraction in rats [153] (**D**); and across all three species (**E**) that are annotated to reside at the mitochondrion. Phosphoproteins from all comparisons shown in Fig. 6 are listed in Supplementary Table S1

In contrast to mitochondrial phosphoproteins, there were a substantially larger number of exercise-regulated phosphosites within the available phosphoproteomic datasets annotated to reside in the nucleus (Fig. 6, Supplementary Table S1; 616, 280 and 99 phosphoproteins in humans, mice and rats, respectively). The proportion of nuclear-annotated phosphoproteins regulated by exercise and/or muscle contraction was relatively consistent between species, ranging between 28 and 38% in analyses of exercise-regulated signalling in human (Fig. 6A) and rodent (Fig. 6C, D, Supplementary Table S1) skeletal muscle. Of the 616 total human muscle exercise-regulated phosphoproteins annotated to the nucleus, 81 were common to the three exercise modalities (Fig. 6B), with a large proportion related to regulation of RNA transcription and apoptotic processes. While differences in the number of exercise-regulated nuclear annotated phosphoproteins between exercise modalities are less pronounced than the mitochondrial compartment, moderate and high-intensity EX were still found to regulate more nuclear phosphoproteins compared with SIE. Contrary to the findings from this post-hoc subcellular compartmental analysis, whole muscle phosphoproteomic analysis across exercise modalities [156] detected a higher number of phosphoproteins regulated by SIE versus EX modalities, suggesting that SIE potentially stimulates more robust responses in compartments other than the mitochondria and nucleus (i.e. cytosol and contractile apparatus). This observation is consistent with findings that failed to detect significant differences in exercise-induced increases in total nuclear p53 protein content following MICE and SIE [142], suggesting that exercise intensity may not have a robust influence on nuclear signalling. However, little research to date has specifically investigated the effects of different exercise volumes and/or intensities on human skeletal muscle subcellular signalling.

Of the > 500 exercise-regulated phosphoproteins annotated to the nucleus across human, mouse and rat muscle, only 49 were found to be regulated across all three species (Fig. 6E). This list of conserved exercise-regulated nuclear phosphoproteins included EX-regulated kinases, such as AMPK and ribosomal S6 kinase. Additionally, TFEB, which controls the expression of mitophagy-related genes and activates PGC-1α following exercise, was regulated and annotated at the nucleus across all species, further implicating TFEB as an important regulator of mitochondrial biogenesis [163]. Collectively, these findings across exercise phosphoproteomic datasets and species provide a greater understanding of the known nuclear and/or mitochondrial protein signalling machinery, and their subcellular localisation and/or potential exercise-responsive translocation between these compartments in whole skeletal muscle samples. These observations also highlight how future skeletal muscle subcellular analyses are warranted to capture the response of more mitochondrial and nuclear proteins to exercise that may ultimately influence mitochondrial biogenesis.

## Future Trends and Directions

There is a growing body of research revealing thousands of phosphorylation sites regulated in response to a single bout of EX in skeletal muscle with respect to research participants (including sex differences), their health status, dietary state and the exercise intervention(s) being utilised [33]. This review has highlighted how protein phosphorylation not only involves changes to a given protein substrate’s structure but also affects their protein–protein interactions, translocation, and activation state. To characterise the dynamic changes in protein phosphorylation and their influence in the regulation of various biological responses to various modes of exercise, MS-based phosphoproteomics offers a unique tool to further identify how proteins change spatially within a tissue type. Recently MS-based phosphoproteomics has been applied to investigate a variety of tissues and cell types and drug treatments, such as β-adrenergic agonist (i.e. forskolin) and calcium agonist (i.e. ionomycin) treatment of cultured rat L6 myotubes to mimic key exercise-related signalling pathways [164] and β-adrenergic receptor agonists and antagonists to study cardiomyocyte contractility [165], leading to the identification of hundreds of site-specific phosphorylation events that may represent new therapeutic entry points for the treatment of cardiomyopathies. Applications of phosphoproteomics have also been extended to understanding insulin signalling [36], in addition to research uncovering AMPK substrates and their potential roles in disease treatment [159] via identification of novel components of the AMPK signalling network. Additionally, commonly used models of muscle contraction have employed phosphoproteomic approaches, providing enhanced mechanistic explanation of mechanotransduction by identifying 663 phosphorylation sites regulated by contraction predominantly via the MAPKs, CAMKs and mTOR pathways [166]. Analyses of pre-existing datasets have also been employed in an attempt to link human phenotype variance in skeletal muscle to personalised interventions, such as exercise and insulin treatment [167]. As such, MS-based approaches offer a powerful tool for future research to uncover personalised and biologically relevant interactions in signalling networks with potential future applications for therapeutic development.

Research technologies, including next generation protein sequencing, are continually advancing to help identify deeper coverage and spatial protein resolution at a subcellular level. Many of these techniques rely on molecular methodologies but lack throughput and the ability to provide proteome-wide information on protein subcellular localisation. However, recently developed spatial MS-based proteomic approaches permit global identification of how proteins change in their native tissue context while revealing spatial differences [168]. One strategy includes matrix assisted laser desorption ionisation (MALDI) imaging mass spectrometry, whereby visualisation of a specific protein or its PTM can be highlighted within a specific tissue type [169]. Future research incorporating subcellular fractionation workflows and/or spatial analyses with MS-based phosphoproteomic approaches has the potential to extend our knowledge of exercise signalling and the framework required to stimulate mitochondrial biogenesis beyond measuring phosphorylation sites in whole tissues, such as skeletal muscle [170]. For example, recent studies have applied subcellular fractionation in mouse skeletal muscle tissue to investigate spatial stress signalling, identifying the relocation of ribosomal proteins in response to muscle contraction [168]. Spatial proteomics and pure mitochondrial fractions from human embryonic kidney (HEK) cells have been utilised to identify > 1100 mitochondrial proteins, generating an important resource of high-confidence mitochondrial proteins termed MitoCoP [171]. In a parallel manner, many proteins reside and/or translocate into the nucleus [172] and the isolation of nuclei by subcellular fractionation has been used to characterise 1174 nuclear proteins and a number of unique phosphorylated proteins associated with apoptosis [173]. The utility of subcellular proteomics has also been demonstrated in the context of mouse liver, with recent research identifying novel nuclear protein and phosphorylation dynamics occurring with the circadian clock [174]. However, the breadth of protein dynamics within the subcellular proteome occurring in human and rodent muscle in response to acute EX remains unexplored.

## Conclusions

The application of phosphoproteomics to interrogate exercise biology has facilitated accurate identification and quantification of the intricate exercise-regulated signalling network responses that occur within skeletal muscle. Few studies to date have investigated acute EX-induced changes in skeletal muscle using phosphoproteomics [153, 154, 156, 157] with relatively small sample sizes in human, mouse and rat models [153, 154, 156, 157]. Considering exercise induces a range of PTMs beyond phosphorylation, the utilisation of other MS-based PTM-omic approaches to quantify and universally map PTM signal transduction (e.g. ubiquitylation [175]) in response to acute exercise is still in its early stages and to date has primarily focused on phosphorylation. Recent developments aimed at streamlining phosphoproteomic sample preparation workflows will permit larger-scale studies and help make these technologies more widely accessible to exercise biologists. Furthermore, large consortia, such as the Molecular Transducers of Physical Activity Consortium (MoTrPAC) [176] and Wu Tsai Alliance [177], have been established to coordinate large-scale efforts to map the molecular responses to exercise across multiple institutions involving omics-based approaches, human cohorts and complementary cell and animal model systems. Only a small fraction of the exercise-regulated skeletal muscle phosphoproteome involves the most widely studied kinases and their substrates, suggesting that there are many unexplored and yet potentially important functional regulatory nodes underlying the wide health benefits of exercise and specifically mitochondrial biogenesis. Future investigations building upon available datasets and utilising both subcellular and omics analysis pipelines will dramatically expand the understanding of exercise signalling networks in the decades ahead.

### Supplementary Information

Below is the link to the electronic supplementary material.

Supplementary file1 (XLSX 57 KB)

## Declarations

### Availability Statement

All data generated or analysed during this study are included in this published article and its supplementary information files.
